# Mathematical modeling in cancer nanomedicine: a review

**DOI:** 10.1007/s10544-019-0380-2

**Published:** 2019-04-04

**Authors:** Prashant Dogra, Joseph D. Butner, Yao-li Chuang, Sergio Caserta, Shreya Goel, C. Jeffrey Brinker, Vittorio Cristini, Zhihui Wang

**Affiliations:** 1 Mathematics in Medicine Program, The Houston Methodist Research Institute, HMRI R8-122, 6670 Bertner Ave, Houston, TX 77030 USA; 2 Department of Mathematics, California State University, Northridge, CA 91330 USA; 3 Department of Chemical Materials and Industrial Production Engineering, University of Naples Federico II, 80125 Naples, Italy; 4 Department of Nanomedicine, Houston Methodist Research Institute, Houston, TX 77030 USA; 5 Center for Micro-Engineered Materials, University of New Mexico, Albuquerque, NM 87131 USA; 6 Chemical and Biological Engineering, University of New Mexico, Albuquerque, NM 87131 USA; 7 Cancer Research and Treatment Center, Molecular Genetics and Microbiology, University of New Mexico, Albuquerque, NM 87131 USA; 8 Self-Assembled Materials Department, Sandia National Laboratories, Albuquerque, NM 87185 USA; 9 Department of Imaging Physics, University of Texas MD Anderson Cancer Center, Houston, TX 78701 USA

**Keywords:** Agent-based modeling, Cancer treatment, Drug transport, Mechanistic modeling, Multiscale, Pharmacokinetics and pharmacodynamics

## Abstract

Cancer continues to be among the leading healthcare problems worldwide, and efforts continue not just to find better drugs, but also better drug delivery methods. The need for delivering cytotoxic agents selectively to cancerous cells, for improved safety and efficacy, has triggered the application of nanotechnology in medicine. This effort has provided drug delivery systems that can potentially revolutionize cancer treatment. Nanocarriers, due to their capacity for targeted drug delivery, can shift the balance of cytotoxicity from healthy to cancerous cells. The field of cancer nanomedicine has made significant progress, but challenges remain that impede its clinical translation. Several biophysical barriers to the transport of nanocarriers to the tumor exist, and a much deeper understanding of nano-bio interactions is necessary to change the status quo. Mathematical modeling has been instrumental in improving our understanding of the physicochemical and physiological underpinnings of nanomaterial behavior in biological systems. Here, we present a comprehensive review of literature on mathematical modeling works that have been and are being employed towards a better understanding of nano-bio interactions for improved tumor delivery efficacy.

## Introduction

**Fig. 1:**
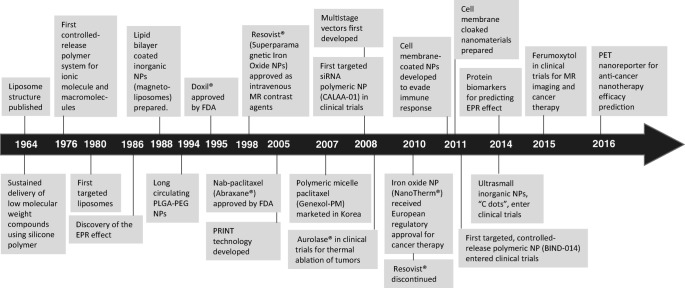
**Timeline**. A historical timeline of the major advancements in cancer nanomedicine (Shi et al. 2016; Hassan et al. 2017)

Leveraging on the improvements in nanotechnology, nanomaterials can be meticulously engineered to obtain reproducible and custom biological behavior, which has catalyzed progress in the field of cancer nanomedicine through targeted delivery of biopharmaceutical agents to solid tumors. Nanoparticle^1^ (NP)-based formulations (nanocarriers) are used to package and deliver cargos that are too toxic, insoluble, rapidly cleared, or unstable for delivery as free molecules (e.g., chemotherapeutic drugs, silencing RNAs, and contrast agents). Nanocarriers have been demonstrated to facilitate the delivery of such agents specifically to the tumor site by *passive accumulation* or *active targeting* (Sykes et al. 2014a), thereby overcoming potential drug-resistance mechanisms (Brocato et al. 2014). Whereas the former phenomenon depends on the leakiness of tumor vessels (defined as enhanced permeability and retention or EPR effect) (Jain and Stylianopoulos 2010), the latter involves surface functionalization of NPs with ligands specific to receptors on the tumor cell surface. A prolonged circulation half-life is generally regarded as a common prerequisite for either process, and NPs have been demonstrated to alter the pharmacokinetics (PK) of the cargo and enhance its circulation half-life, thereby promoting delivery specifically to the tumor and reducing collateral damage to healthy cells (Chow et al. 2011). Additionally, nanocarriers can be engineered to allow triggered release of cargo, i.e., the payload remains encapsulated inside NPs unless a particular physiological or external stimulus is presented, e.g., pH, enzymes, temperature, or electromagnetic radiation. To date, a diverse variety of nanomaterials have been developed for tumor delivery applications. They include organic (e.g., polymeric, liposomal, solid lipid) and inorganic (e.g., silica, gold, iron, titanium) NPs, which can be spherical, rod-like, or disc-like in shape (among others), can be synthesized in a range of sizes, and possess a net surface charge (zeta potential). While remarkable progress has been made in the field of cancer nanomedicine over the past 50 years (Fig. 1), and some nanocarriers are already in clinical use (Table 1), there are significant challenges that remain unanswered, impeding the clinical translation of a majority of nanocarriers (Shi et al. 2016).

**Table 1:** Anti-cancer nanomedicines in the clinic

Drug/Agent	Formulation	Product name/Company	Applications	NP size (nm)	Half-life (h)	References
Asparaginase	Polymeric conjugates	Oncaspar® (PEG^a^)/Baxalta	Acute lymphoblastic leukemia	50–200	145–189.6	(Douer et al. 2007; Bawa et al. 2016)
Cytarbine	Liposome	Depocyt®/Pacira Pharmaceuticals	Lymphomatous leukemia	10–20 × 10^3^	82.4	(Pillai 2014; Bulbake et al. 2017)
Daunorubicin	Liposome	DaunoXome®/Ga-len	HIV-related Kaposi’s sarcoma	45	4–5.6	(Fumagalli et al. 2000; Bellott et al. 2001; Immordino et al. 2006; Bulbake et al. 2017; Praça et al. 2017)
Doxorubicin	Liposome	Doxil®/ Caelyx®/Janssen	Kaposi’s sarcoma Multiple Myeloma Ovarian cancer	87.3 ± 8.5	50–60	(Gabizon et al. 1993; Gabizon 2001; Gabizon et al. 2003; Gabizon et al. 2006; Soundararajan et al. 2009)
	Liposome	Myocet®/Teva UK	Metastatic breast cancer	150–250	2–3	(Immordino et al. 2006; Bulbake et al. 2017)
Irinotecan	Liposome	Onivyde®/Merri-mack Pharmaceuticals	Metastatic pancreatic cancer	110	19.4–22.3	(Drummond et al. 2006; Agency 2016)
Iron oxide	SPION^b^	NanoTherm®/M-ag-Force AG	Glioblastoma	12–20	N/A	(Bellizzi and Bucci 2018)
Mifamurtide	Liposome	Mepact®/Takeda Pharmaceutical	Osteocarcoma	1–5 × 10^3^	2.03–2.27	(Ando et al. 2011; Venkatakrishnan et al. 2014; Yu and Li 2014)
Paclitaxel	Polymeric micelles	Genexol®-PM/Samyang Biopharmaceutic-als	Non-small cell lung cancer Breast cancer Ovarian cancer	20–50	11–12.7	(Kim et al. 2004; Werner et al. 2013)
Paclitaxel	Albumin-bound	Abraxane®/Celg-ene	Advanced metastatic pancreatic cancer Advanced metastatic breast cancer Advanced non-small cell lung cancer	130	20.5–21.6	(Di Costanzo et al. 2009; Miele et al. 2009)
Styrine maleic anhydride neocarzinostatin	Polymer protein conjugate	Zinostatin Stimalamer	Unresectable hepatocellular carcinoma	N/A	7.1	(Toge et al. 1991)
Vincristine	Liposome	Marqibo®/Spectr-um Pharmaceuticals	Acute lymphoblastic leukemia	90–140	2.4–4.9	(Talon Therapeutics, Inc., 2012; Fenske and Cullis 2008; Silverman and Deitcher 2013; Yu and Li 2014)

^a^PEG (polyethylene glycol) is a hydrophilic polymer that is used to preclude plasma protein adsorption on NP surface through steric hindrance

^b^Superparamagnetic iron oxide nanoparticles

**Fig. 2:**
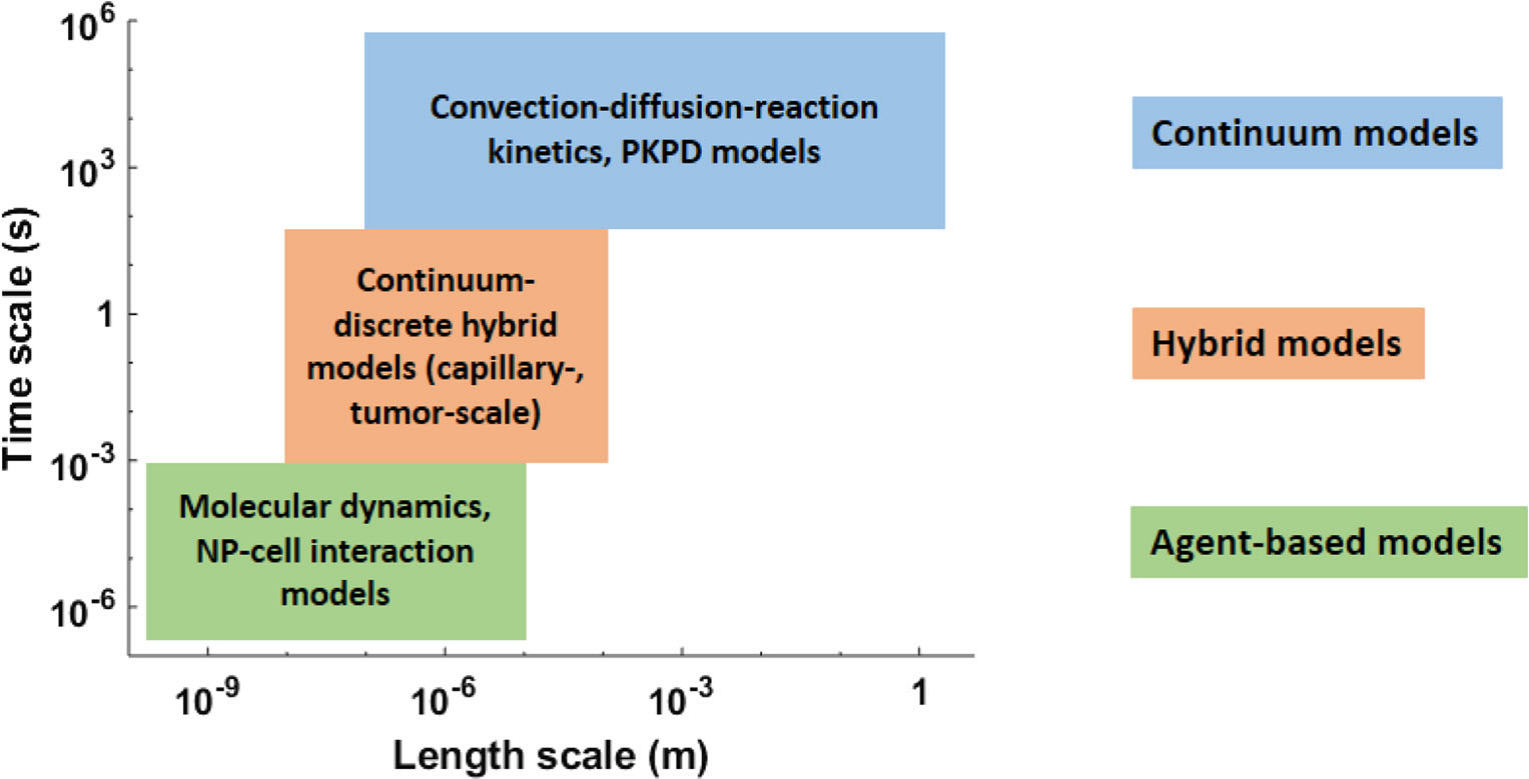
**Classification of mathematical models.** Mathematical models in cancer nanomedicine can be classified based on the characteristic spatiotemporal scale of the system under consideration

Based on a meta-analysis of literature in cancer nanomedicine over the past decade (Wilhelm et al. 2016), it has been noted that on average only 0.7% of the injected dose of NPs reaches the tumor. This is generally ascribable to the *biological barriers to transport of NPs that span across multiple scales and pose a serious threat to the clinical translation of upcoming nanoformulations*, making it imperative to investigate the underlying nano-bio interactions responsible for the suboptimal tumor deliverability of nanocarriers. While certain physicochemical properties of NPs may correlate with higher tumor deliverability in vitro or in vivo (e.g., size <100 nm, neutral charge, and rod-shape (Albanese et al. 2012; Wilhelm et al. 2016)), an improved understanding of biological barriers faced by nanocarriers can provide NP design guidelines for optimized tumor delivery, and foster their clinical translatability. To this end, mathematical modeling has been an important tool in supporting cancer nanomedicine, and also for many other cancer research fields (Wang and Deisboeck 2014; Wang et al. 2015; Wang and Maini 2017). For example, modeling has provided mechanistic understanding of phenomenological observations based on physical principles and helped establish important quantitative relationships. Modeling efforts in cancer nanomedicine have been applied to phenomena spanning several biologically relevant scales in time and space (Fig. 2). Here, we review various mathematical modeling techniques that have been helpful to the field of cancer nanomedicine to answer questions related to nano-bio interactions and to provide insights into the problem of low tumor deliverability of NPs.

## The voyage of nanoparticles

**Fig. 3:**
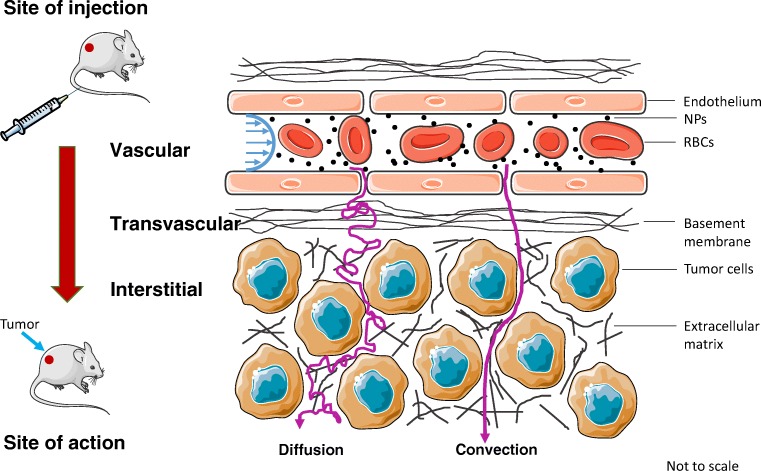
**Nanoparticle transport in tumors.** Biophysical barriers involved in the delivery of NPs to tumors via microcirculation

Before we discuss any specific modeling work, it is critical to understand the journey of NPs from the site of injection to the site of action, which can be broadly divided into three phases: i) vascular, ii) transvascular, and iii) interstitial, to reach the cancerous cells (Fig. 3) (Jain and Stylianopoulos 2010; Chauhan et al. 2011; Nichols and Bae 2012). NPs are faced with physical and physiological challenges during these stages of transport that strongly influence their biological fate.

Immediately after NPs are injected into the blood stream, they are exposed to a high concentration of plasma proteins (60–80 g ∙ l^−1^), such as albumins, apolipoproteins, and opsonins, which adsorb on the particle surface and form a surrounding biomolecular corona that redefines their chemical identity and provides them with the so-called *biological identity*, and ultimately plays an important role in governing nano-bio interactions (Monopoli et al. 2012; Caracciolo et al. 2017). The bimolecular corona around the particles is a dynamic entity and, depending upon the relative affinity of the proteins for the NP surface, the coronas at steady state can have varied compositions. The nature of the corona can impact nano-bio interactions by affecting the hydrodynamic size, surface charge, and immunogenicity of the NPs, thereby affecting their cellular internalization, biodistribution, and circulation half-life (Aggarwal et al. 2009).

Simultaneously, NPs are transported across the body via the vascular network, and upon arrival at the finest blood vessels, i.e., capillaries, particles are faced with special anatomical, physiological, and hemodynamic conditions that strongly influence their fate. Healthy capillaries are broadly classified as: i) continuous, ii) fenestrated, and iii) sinusoidal, depending upon the upper limit of pore size in the vessel walls. Continuous capillaries have pore sizes <5 nm (e.g., brain, lungs, muscles, skin), fenestrated capillaries haves pores <15 nm (e.g., kidneys), and sinusoids in liver have pores <200 nm, while those in spleen are ~5 μm (Sarin 2010). Thus, the NP to pore size ratio becomes a determining factor (besides surface charge) in the extravasation of NPs into tissue interstitium (Stylianopoulos et al. 2013) or excretion in kidneys (Choi et al. 2007). In addition, the presence of resident macrophages in the lumen of capillaries causes NPs, which are already opsonized,^2^ to be imminently phagocytosed and removed from circulation, thereby affecting their circulation time in the body. Kupffer cells of the liver (Tsoi et al. 2016) and splenic macrophages (Cataldi et al. 2017) have been recognized to contribute significantly to this mechanism of NP clearance. Given the significant decrease of blood flowrate in capillaries (~1 mm ∙ s^−1^), compared to larger vessels (>10 cm ∙ s^−1^), hemodynamic conditions exist in capillaries conducive for NP interaction with vessel wall pores or immune cells (Jain and Stylianopoulos 2010; Tsoi et al. 2016). However, it is important as a prerequisite that NPs gain near-wall access via a size-, shape-, and hematocrit^3^-dependent phenomenon referred as *margination*^4^ (Lee et al. 2013; Müller et al. 2014). As a result, a complex interplay between these microscopic interactions inside blood capillaries defines the global biodistribution and clearance of NPs from the body, thereby strongly influencing the tumor delivery of NPs (Dogra et al. 2018).

Once NPs enter into the tumor interstitium following capillary extravasation, they are subject to a hostile microenvironment that weakens convective transport; thus diffusion becomes the primary means of transport for NPs. This significantly limits the penetration distance and delivery of cargo to cancerous cells distant from the tumor-feeding capillaries (Deisboeck et al. 2011; Pascal et al. 2013b; Wang et al. 2016; Cristini et al. 2017). *Readers are referred to the following review for a detailed discussion on intra-tumoral transport barriers responsible for chemotherapy resistance* (Brocato et al. 2014).

## Mathematical modeling in cancer nanomedicine

We now discuss mathematical modeling works that focus on the above mentioned biophysical processes for optimizing NP design towards improved tumor delivery and efficacy. The following sections are organized based on the problem they seek to investigate (Table 2). Upon entering the blood stream, the *formation of an enveloping protein corona* changes the biochemical properties of NPs, a process which is best described mathematically by kinetic models or coarse-grained molecular dynamics simulations. Subsequent to corona formation, the biochemically altered NPs are transported via blood to tumor and various organs. The *processes during transport* are modeled by discrete, hybrid, or continuum models at the microscopic or mesoscopic length scales. Once NPs arrive at the capillary wall or extravasate into the extravascular space, *cellular uptake* of NPs is modeled using discrete modeling approaches. The emergent *whole-body distribution and clearance* of NPs due to microscopic nano-bio interactions is described by pharmacokinetic models, while *tumor deliverability* of particles is studied using hybrid modeling methods. Finally, *nanotherapy efficacy and toxicity* are evaluated with pharmacodynamic models.

**Table 2:** A summary of key mathematical modeling approaches in cancer nanomedicine

Biological problem	Modeling-type	References	Major findings	Clinical relevance
Biomolecular corona formation	Kinetic modeling	(Dell'Orco et al. 2010; Dell'Orco et al. 2012; Sahneh et al. 2013)	During corona formation, high affinity proteins displace low affinity proteins (Vroman effect), and the corona evolves from a metastable to a stable state. NP size is more important than number and size of peptides bound to NP surface in governing successful NP-cell surface receptor binding.	Provide a framework to study microscopic nano-bio interactions in various physiological conditions.
	Coarse-grained molecular dynamics simulations	(Lopez and Lobaskin 2015; Tavanti et al. 2015a)	Protein adsorption energies for NP-protein interaction are primarily affected by NP size, while surface charge only has a small effect.	
Microvascular transport, margination, and binding	Continuum modeling	(Gentile et al. 2008)	An increase in hematocrit or vessel permeability reduces the effective diffusion coefficient of NPs, highlighting implications to intravascular transport of NPs.	Provide insights into capillary-scale biophysical interactions of NPs that can impact their macroscopic behavior, thereby providing design guidelines to optimize systemic circulation kinetics.
		(Tsoi et al. 2016)	NP sequestration in liver sinusoid is jointly affected by hemodynamic conditions and NP characteristics.	
	Hybrid modeling	(Lee et al. 2013; Müller et al. 2014; Fullstone et al. 2015)	Larger NP size correlates with greater margination, which is further promoted by discoidal NP shape and higher hematocrit.	
Cellular internalization	Discrete modeling	(Gao et al. 2005; Decuzzi and Ferrari 2008; Yuan and Zhang 2010)	A minimal particle size and ligand density are necessary for effective endocytosis.	Provide mechanistic understanding of the cellular uptake of NPs, which has implications in drug delivery or NP clearance by immune cells.
Whole-body biodistribution and clearance	PK modeling	(Dogra et al. 2018)	Small NP size correlates with longer systemic circulation and lower accumulation in mononuclear phagocytic system (MPS) organs, irrespective of route of injection. Positive charge supports excretion, and surface exposure of charged molecules increases the vulnerability to sequestration in MPS organs.	Provide a mechanistic description of whole-body phenomenological observations important for quantifying structure-activity relationships of NPs.
Tumor deliverability	Hybrid modeling	(Chauhan et al. 2012; Hendriks et al. 2012; Frieboes et al. 2013; Stapleton et al. 2013; Sykes et al. 2016)	Interplay between NP physicochemical properties (especially, size, surface charge) and vascular characteristics affects EPR-based accumulation and delivery of NPs to cancerous cells in the tumor interstitium.	These models provide insight on the intra-tumoral transport of NPs and provide critical design guidelines for improved tumor deliverability.
Nanotherapy efficacy and toxicity	PD modeling	(Pascal et al. 2013a; Leonard et al. 2016; Wang et al. 2016; Miller and Frieboes 2018)	Time integrated NP uptake by cancerous cells governs therapy efficacy. The outcome however is non-trivially affected by patient-specific tumor-perfusion heterogeneities.	Provide predictive tools that can be employed prospectively in the clinic to design personalized-nanomedicine regimens.
		(Laomettachit et al. 2017)	NP toxicity to healthy liver cells is dose-dependent, and while the effect of small exposures can be reversed due to cell proliferation, tissue damage due to higher dose exposures are generally irreversible.	Such studies are critical in assessing the toxicity potential of nanocarries in clinical doses, thereby providing guidelines for safe exposure limits.

### Biomolecular corona formation

#### Kinetic modeling

In a first attempt to model the NP-protein interactions during corona formation, Dell’Orco et al. developed a mathematical model to describe the kinetics of competitive interaction of human serum albumin (HSA), high density lipoprotein (HDL), and fibrinogen for binding with a 70 nm-sized copolymer NP (Dell'Orco et al. 2010). The model, based on the law of mass action, is formalized as a system of three linear first order differential equations describing the interaction of each protein with the NPs, represented as:

1 $$ \frac{d\left[ NP\bullet pro\right]}{dt}={n}_{pro}{k}_{pro}^{on}\left[ NP\right]\left[ pro\right]-{k}_{pro}^{off}\left[ NP\bullet pro\right], $$

where [*NP*], [*pro*], and [*NP* ∙ *pro*] represent the concentration of NPs, protein, and NP-protein complex, respectively, and ${k}_{pro}^{on}$ and ${k}_{pro}^{off}$ are the association and dissociation rate constants of NP-protein binding. *n*_*pro*_, which is the number of available binding sites on NP surface for a given NP-protein pairing, is approximated by the ratio of surface area of the NP-protein complex and cross-sectional area of protein molecules, assuming a spherical shape, given by ${n}_{pro}=\frac{4\pi {\left({r}_{NP}+{r}_{pro}\right)}^2}{\pi {r}_{pro}^2}$ protein radius. This model has been used to simulate the temporal evolution of the corona composition that seems to depend on the association and dissociation rate constants of the proteins that govern the time required to reach steady state. Simulation results show that over time, the high affinity HDL displaces HSA from the corona, despite the much higher plasma concentration of the latter, primarily because of a relatively lower affinity of HSA for the NPs (Vroman effect). They also extended the model to study the effect of corona formation on the binding of NPs to target cell surface receptors, taking into account the kinetic, stoichiometric, and geometric variables of the system (Dell'Orco et al. 2012). Global sensitivity analysis revealed that successful NP-receptor binding is more strongly dependent on NP size than the number and size of peptides bound to NP surface.

Sahneh et al. made further improvements to the model by using population balance equations to derive the ordinary differential equations (ODEs) for *successive* binding of HSA, HDL, and fibrinogen to NPs, i.e. binding of a protein when the NP is already covered with different proteins, thereby representing a more realistic physiological scenario (Sahneh et al. 2013). Briefly, Sahneh et al. revised the NP-protein binding biochemical reactions such that Eq. (1) is modified to:

2 $$ \frac{d\left[ NP\bullet pro\right]}{dt}={n}_{pro}{k}_{pro}^{on}\left[ pro\right]Y-{k}_{pro}^{off}\left[ NP\bullet pro\right] $$

where $Y=\left({\left[ NP\right]}_0-\sum \limits_{j=1}^m\frac{\left[{pro}_j\bullet NP\right]}{n_{pro_j}}\right)$ is the concentration of available binding sites, as governed by the presence of already bound proteins on NP surface. This model reveals the temporal evolution of corona formation progressing from an early *metastable* composition to a late *stable* composition representing the hard corona, thus providing a mathematical underpinning to the phenomenon which can be used to make projections about NP behavior in varied physiological conditions. As a further extension of these modeling efforts, Zhdanov and Cho incorporated irreversible protein reconfiguration (denaturation) into the kinetic equations of adsorption and desorption of proteins on the NP surface (Zhdanov and Cho 2016). They also studied the role of protein diffusion during the adsorption process, given that the diffusion rate constant diminishes at the solution-solid interface compared to the bulk, and ultimately revealed that the rate limitations imposed by protein diffusion are negligible.

In the models discussed above, independent, single binding sites are assumed such that protein-protein interactions and cooperative effects are considered insignificant. To investigate this assumption, Angioletti-Uberti et al. developed a model based on the dynamic density functional theory (DDFT) which describes the density evolution of systems undergoing Brownian dynamics, to include the effects of inter-particle interactions on the adsorption kinetics of proteins (Angioletti-Uberti et al. 2014). Specifically, the free-energy function in their DDFT model is composed of the translational free energy of the proteins, free energy of protein-NP interaction, and free energy of protein-protein interactions. The model was used to understand the temporal evolution of the density profile of proteins around the NP-solution interface and protein adsorption on NP surface. In comparison to a reference model with ideal diffusion alone and no protein interactions, this model resulted in closer agreement with experimental findings. Thus, by incorporating fluxes due to different chemical potential gradients in the system, DDFT gives a more comprehensive picture than a simple diffusion-based model.

#### Coarse-grained molecular dynamics simulations

**Fig. 4:**
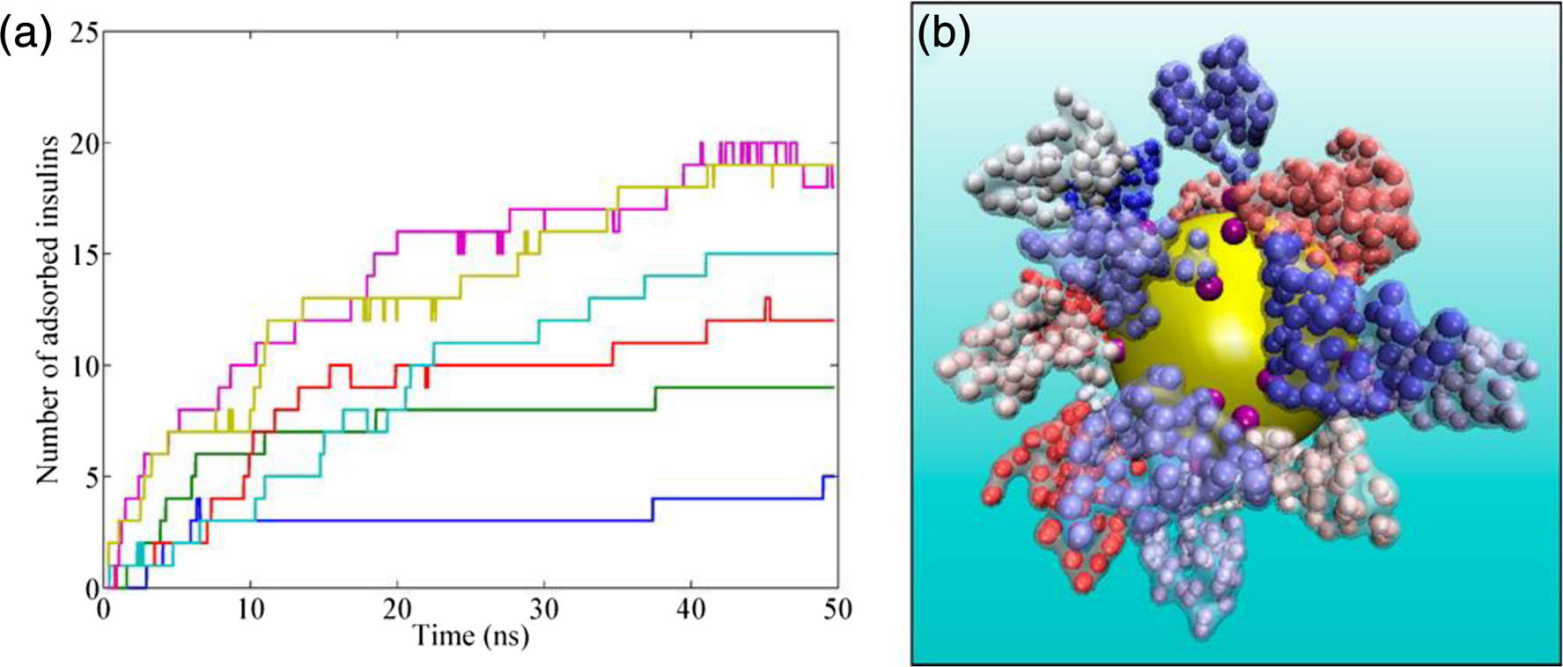
**Biomolecular corona formation studied with CG modeling. a** Moving average (25 time steps; each time step represents 1 fs in real time) of number of insulin proteins adsorbed on citrate-coated gold NP over time. Colors represent different number of insulin molecules in the solution: blue, 10; green, 20; red, 34; cyan, 50; purple, 70; and brown, 100. **b** Snapshot, from a model simulation of a NP with 70 insulin molecules in solution taken at 45 ns shows the surrounding corona formation. Reproduced with permission from (Tavanti et al. 2015b)

While advancement of technology has made great strides to expand the ability to computationally simulate the atomic interactions of biochemical processes involving a handful of proteins for several nanoseconds, the feasibly-modeled size and time scales for such all-atom models are still well below what is relevant to the formation of a biomolecular corona around a NP (Tozzini 2005; Lopez and Lobaskin 2015). To reduce the degrees of freedom so that simulations of corona formation can be effectively carried out, coarse-graining techniques (CG) have been developed and employed in recent computational studies (Fig. 4). The CG approximation has been previously adopted to simulate phenomena associated with protein adsorption onto flat surfaces, such as the Vroman effect (Vilaseca et al. 2013). In a protein, atoms within each amino acid may be coarse-grained and thus represented by one or a few beads, allowing the interconnecting force fields between these beads to be incorporated. There are two major classes of CG protein models: elastic network models and Go-type models. In an elastic network model, the force fields are approximated as springs between beads, suitable for including thermal fluctuations and analyzing principal modes (Tozzini 2005). For corona formation, thermal fluctuations are negligible compared to the adsorption energies and ruggedness of protein folding energy landscapes, and Go-type models are usually adopted (Tozzini 2005; Tavanti et al. 2015a; Shao and Hall 2016). In a canonical Go model, a bead is placed at the α-carbon position of each amino acid residue that collectively forms the crystal structure of a protein. Such a “one-bead” model can further be refined by placing one or more beads to represent the positions of the side chains if more details of the protein structure are desired and computationally feasible. The simulation of corona formation is carried out by discontinuous molecular dynamics, for which the protein structure can be retrieved from the Protein Data Bank (PDB) (Berman et al. 2003), and force fields governing the dynamics are evaluated between adjacent amino acid residues and between the surface of NPs through CG models. Typically, the force fields, expressed as interaction energies, consist of (Tavanti et al. 2015a, b)

3 $$ U=\sum {U}_{\mathrm{bonded}}+\sum {U}_{\mathrm{non}-\mathrm{bonded}}, $$

where the bonded force fields are

4 $$ \sum {U}_{\mathrm{bonded}}=\sum \limits_{i,j}{U}_{\mathrm{bonds}}\left(i,j\right)+\sum \limits_{i,j,k}{U}_{\mathrm{angles}}\left(i,j,k\right)+\sum \limits_{i,j,k,l}{U}_{\mathrm{dihedrals}}\left(i,j,k,l\right). $$

The first term on the right-hand side is the bonding energy between two consecutive amino acids; the second term describes the bending energy due to the angle between two consecutive bonds, hence three consecutive amino acids; and the last term describes the bending energy due to the dihedral angle of four consecutive amino acids. The non-bonded forces typically involve van der Waals interactions *U*_VdW_ and electrostatic interactions *U*_el_ between two arbitrary non-bonded amino acids:

5 $$ \sum {U}_{\mathrm{non}-\mathrm{bonded}}=\sum \limits_{i,j}{U}_{\mathrm{VdW}}\left(i,j\right)+\sum \limits_{i,j}{U}_{\mathrm{el}}\left(i,j\right), $$

based on the pairwise Hamaker approach. For the process of corona formation, the interactions between amino acids and the surface segments of NPs are described by both bonded and non-bonded interactions.

Based on the concept of CG models, Lopez and Lobaskin studied the adsorption of six common blood plasma proteins onto hydrophobic NPs (Lopez and Lobaskin 2015). They showed that the size of the NP, and thus the curvature of the NP surface, affected the adsorption energies considerably for different protein orientations. Comparatively, NP surface charges had small effect on adsorption energies. Tavanti et al. used a CG model to investigate the adsorption of ubiquitin onto the surface of gold NPs for various NP sizes and coating conditions of NP surfaces (Tavanti et al. 2015a). They observed a two-phase corona formation, where a consistent number of proteins (depending on NP size) were quickly attracted to the NP surface in a few nanoseconds; however, the final configuration of the corona was not achieved until a slow reorientation, which could even be prolonged if NPs were coated with citrates, as ubiquitin competed with citrates for binding sites. Similar competition was also observed in the CG simulations between the adsorption of insulin and fibrinogen onto gold NPs (Tavanti et al. 2015b). In such models, force field parameters may be experimentally measured using monocomponent solutions of each protein. For example, Vilanova et al. parameterized their model by individually evaluating the binding affinities of three proteins, HSA, transferrin, and fibrinogen, and their model prediction of the prolonged adsorption due to competition agreed well with experiments (Vilanova et al. 2016). Shao and Hall used CG simulations to examine seven known isothermal adsorption formulas estimating the equilibrium adsorption coverage with varying protein concentration (Shao and Hall 2016). They found that for the two proteins (Trp-cage and WW domain) that they studied, four of the adsorption formulas (Langmuir, Freundlich, Temkin, and Kiselev) described the results reasonably well, while the remaining three (Elovich, Fowler-Guggenheirm, and Hill-de Boer) performed relatively worse.

#### Machine learning

For the purpose of constructing a predictive model of corona formation to guide the engineering of NP designs, machine learning is a conceptually similar approach to coarse-graining in that it trades accuracy for efficiency. Instead of incorporating mechanical descriptions of the physiochemical processes, machine learning *statistically* infers observable features from controllable physiochemical properties. For example, using random forest classification, Findlay et al. constructed a decision tree learning model to predict the corona composition from biophysicochemical properties of proteins, NPs, and the surrounding solution (Findlay et al. 2018). Through model training, they were also able to estimate the weight of each biophysicochemical property, which could potentially be useful for guiding the design of NPs.

### Microvascular transport, margination, and binding

#### Continuum models

Decuzzi and Ferrari et al. studied the longitudinal transport of nanocarriers in non-permeable and permeable capillaries using the Taylor-Aris theory of shear dispersion (Decuzzi et al. 2006; Gentile et al. 2008). The transport of solute under fluid flow is governed by a convection-diffusion equation:

6 $$ \frac{\partial C}{\partial t}={D}_m{\nabla}^2C-u\bullet \nabla C $$

where *C* is the local concentration of the solute, *u* is the fluid velocity field, and *D*_*m*_ is the diffusion coefficient^5^ of the solute. This equation suggests that NPs in blood are transported under the influence of pressure gradient driven blood flow (advection) and their inherent Brownian motion (diffusion). Taylor (Taylor 1953) and Aris (Aris 1956) introduced the concept of an effective diffusion coefficient (*D*_eff_) as a solution to the above equation averaged over the cross-section of a cylindrical tube of radius *R*_*e*_ and mean fluid velocity *U*, thus combining the contribution of both convection and diffusion in the longitudinal dispersion of NPs such that

7 $$ {D}_{\mathrm{eff}}={D}_m\left[1+{P}_e^2/48\right], $$

where *P*_*e*_ is the Peclet number (*P*_*e*_ = *R*_*e*_*U*/*D*_*m*_). Using the Taylor-Aris approach, Decuzzi and Ferrari et al. obtained an analytical formulation for the effective diffusion coefficient of NPs in a blood capillary (approximated as a cylindrical tube), incorporating the effects of vessel permeability and blood rheology. Eq. (7) is thus modified to:

8 $$ {D}_{\mathrm{eff}}={D}_m\left[1+\frac{P_{e_0}^2}{48}\frac{{\left(\cosh \left(\overset{\sim }{z}\Gamma \left({\upxi}_c\right)\right)-\varOmega \cosh \left(\Gamma \left({\upxi}_c\right)\right)-\overset{\sim }{z}\Gamma \left({\upxi}_c\right)\right)}^2\bullet G\left({\upxi}_c\right)}{1-\varOmega \cosh \left(\Gamma \left({\upxi}_c\right)\right)}\bullet G\left({\upxi}_c\right)\right], $$

where ${P}_{e_0}$ is the Peclet number at vessel inlet, $\overset{\sim }{z}$ is the dimensionless longitudinal coordinate, *Г* is the permeability parameter, *Ω* is the pressure parameter, ξ_*c*_ is the rheological parameter (ratio between the plug and the vessel radii assuming a non-Newtonian Casson fluid velocity profile), and *G* is an expression that depends upon ξ_*c*_. With this model, they found that an increase in hematocrit or vessel permeability causes a reduction in the effective diffusion coefficient of NPs, highlighting implications to intravascular transport of NPs.

In order to establish the mechanism of clearance of hard nanomaterials by the liver, Tsoi et al. implemented a minimal model of the liver sinusoid in order to study the role of flow dynamics and NP physicochemical properties on sequestration of NPs in the sinusoids (Tsoi et al. 2016). The sinusoid is modeled as a cylindrical channel of length *L* and radius *r*_0_, with the inner wall of the channel lined by cells capable of sequestering NPs, which move in the channel under the influence of pressure gradient-driven advection along the longitudinal axis and Brownian motion along the radial axis. The model is expressed as a partial differential equation (PDE) that defines the temporal evolution of NP density along the length of the channel. The PDE is then solved to obtain an expression for the probability *P* of NP sequestration in the channel, given by:

9 $$ P=\sum \limits_{i=1}^{\infty}\left[1-\exp \left(-\frac{DL}{U{r}_0^2}{\lambda}_i\right)\right]{b}_i, $$

where *D* is NP diffusivity, *U* is the average flow velocity, and *λ*_*i*_ and *b*_*i*_ are numerical coefficients that depend upon flow profile and boundary conditions. In this expression, $\frac{DL}{U{r}_0^2}$ resembles the inverse of the Peclet number (1/*P*_*e*_) and is the ratio of the rate of diffusion to the rate of advection that controls the extent of NP interaction with the channel walls. Further, to account for the effect of NP-cell pair properties on cellular internalization of NPs and under the assumption that the cells do not function as perfect traps, a sticking coefficient (ratio of dissociation constant to association constant) is introduced into the model, making the model predictions more reliable. Model analysis demonstrated that hemodynamic conditions and particle properties that lead to $\frac{DL}{U{r}_0^2}\gg 1$ favor greater NP sequestration in the channel, explaining the underlying mechanism for high NP sequestration in liver.

#### Discrete models

In order to study the margination of NPs in blood vessels, Decuzzi et al. developed a mathematical model of a spherical NP of radius *R* circulating freely in blood at distance *z* from the endothelial wall (Decuzzi et al. 2005). The model considers buoyancy, hemodynamic, van der Waals, electrostatic, and steric force interactions acting on the particle and takes the form of the following non-linear differential equation:

10 $$ {a}_1\overset{\sim }{h}\left(\eta \right)\frac{d\eta}{d\tau}-\frac{a_3}{{\left(\eta -1\right)}^4}+{a}_2{e}^{-\overset{\sim }{k}\left(\eta -1\right)}+{a}_4{e}^{-\frac{\eta -1}{{\overset{\sim }{R}}_g}}=1, $$

where the parameter *a*_1_ gives the ratio between hemodynamic force and buoyancy; *a*_2_ gives the ratio between the repulsive electrostatic force and buoyancy; *a*_3_ gives the ratio between the attractive van der Waals interaction and buoyancy, and *a*_4_ gives the ratio between the repulsive steric force and buoyancy. *η* is the dimensionless distance of the particle from the endothelial wall, $\overset{\sim }{h}$ accounts for the hemodynamic forces acting on the particle, $\overset{\sim }{k}$ is the dimensionless Debye length, and ${\overset{\sim }{R}}_g$ is the dimensionless radius of gyration of the polymer chains on the NP surface. Numerical solution of the model determines the trajectory of the NP in the blood stream, in addition to its margination velocity and the time needed to contact the endothelium. Although the model did not account for the presence of erythrocytes, it revealed some interesting results. For example, it found that as the size of the particle is reduced, the time needed to reach the wall increases up to a maximum, beyond which the time to reach the wall decreases as the radius is further reduced. NP margination was further explored in a more comprehensive manner by Decuzzi and Lee et al. using a computational model of erythrocyte and NP transport in blood capillaries based on the immersed finite element method (discussed in next section) (Lee et al. 2013).

Furlani and Ng developed an analytical model specifically focused on magnetic NPs, to study their microvascular transport and capture under the influence of an external magnetic field (Furlani and Ng 2006). The model primarily accounts for magnetic and viscous forces acting on the particles and solves for the trajectory of the NPs in microvessels. By inputting magnetic force (obtained by the “effective” dipole moment method^6^) and fluidic force (obtained from Stoke’s law^7^) into Newton’s second law of motion, they arrived at an analytical expression for the trajectory of the NPs:

11 $$ {\overline{x}}^3-3\overline{x}+3f\left({\overline{x}}_0,{\overline{z}}_0,\overline{z}\right)=0, $$

where $\overline{x}$ and $\overline{z}$ are the dimensionless position coordinates of the NP along the *x*- and *z*-axis, respectively; *f* is a function of $\overline{z}$ and the normalized initial position coordinates of the NP ${\overline{x}}_0,{\overline{z}}_0$. Since magnetic force has no *y*-component, the model assumes NP motion in the *x*-*z* plane only. Their model demonstrates the suitability of paramagnetic NPs for delivering payload specifically to tumors under the influence of externally applied magnetic fields. Their model also determined the minimum radius of the particle required for successful capture in the capillary, by showing that the NP critical size is a function of the distance of the magnet from the capillary.

#### Hybrid models

**Fig. 5:**
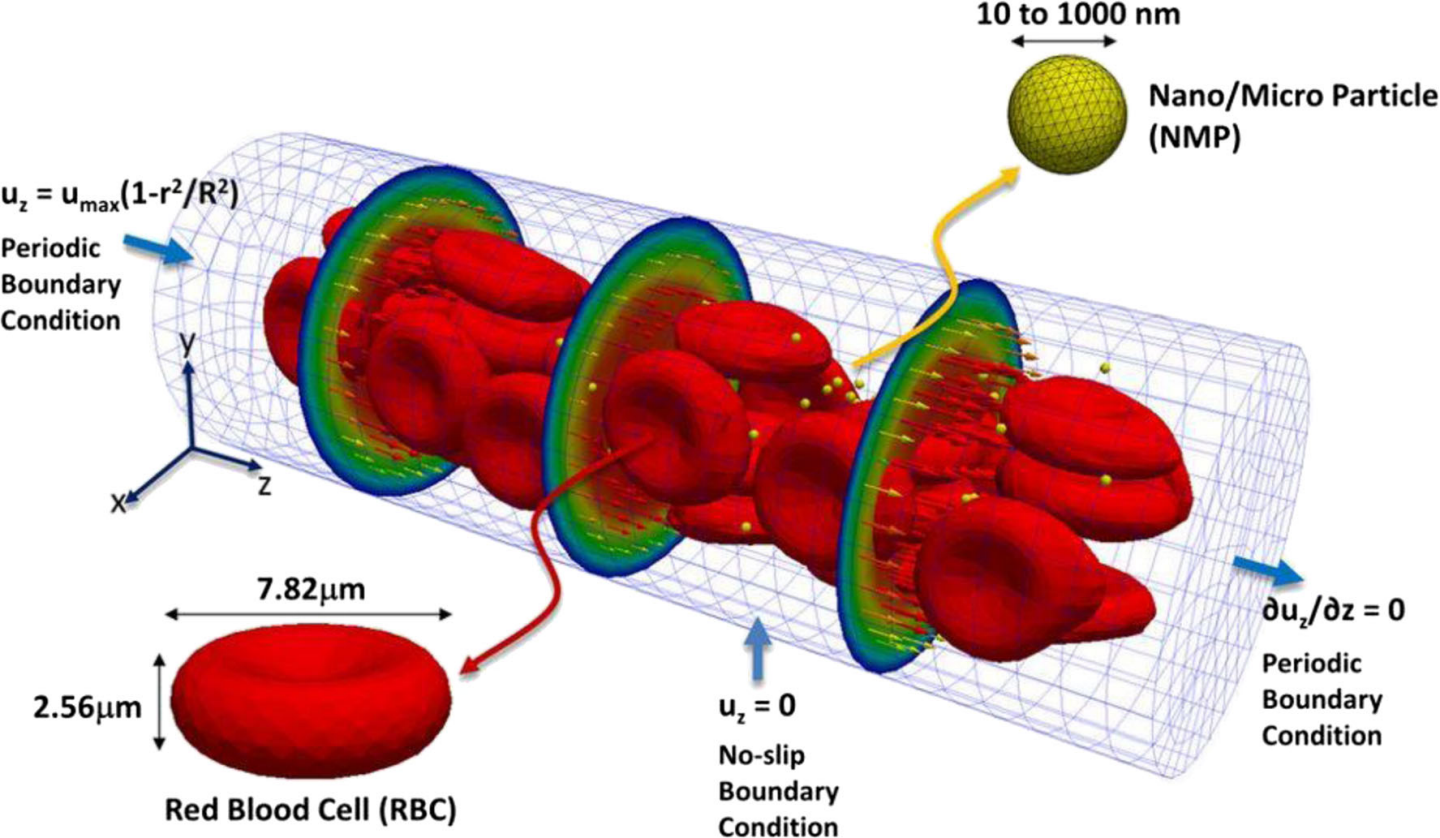
**Computational domain for hydrodynamic simulations of NP transport in microcirculation.** NP transport was studied in a capillary of length 60 μm and diameter 20 μm in the presence of deformable red blood cells. Periodic boundary conditions are imposed at the inlet and outlet of the capillary, and a parabolic velocity profile with a maximum velocity of 100 μm ⋅ s^−1^ is imposed at the inlet. Reproduced with permission from (Lee et al. 2013)

Hydrodynamic modeling hybridized with agent-based descriptions of NPs and blood cells has shed valuable insights into the dynamics of NP movement within capillary flow and shed new light on how NPs may be designed to increase extravasation. This is accomplished through a Navier-Stokes description of blood plasma flow within the capillary, which is solved numerically to obtain flow dynamics around agent representations of blood cells and NPs; these in turn move within the capillary due to forces applied as determined by the solution of the Navier-Stokes equation at their location (Fig. 5). Using these techniques, Lee et al. showed that NPs ≤ 100 nm diameter tended to remain in circulation with the blood bulk and have reduced incidences with and adhesion to the vasculature wall, while particles in the 500–1000 nm range were more likely to be displaced by red blood cells towards the vasculature walls (margination) (Lee et al. 2013). This increased rate of incidence with the vasculature endothelium likely increases extravasation rates and contributes to higher tumor accumulation in particles ≥100 nm than those ≤100 nm. Previously, Lee et al. had also studied the effect of particle shape on the margination propensity in linear laminar flow, although without incorporating RBCs into the model, and found that particles with discoidal shape and low aspect ratio have the highest propensity to marginate (Lee et al. 2009a, 2009b).

Interestingly, Müller et al. found similar effects of particle size on NP radial distribution within the capillary, and further demonstrated that particle shape, hematocrit, and capillary diameter also play key roles in NP distribution and dynamics within the capillary, as well as on NP extravasation (Müller et al. 2014). They found that increased hematocrit, smaller capillary diameter, and NP diameter ≥ 250 nm all promote increased margination; but, unlike the observations of Lee et al. discussed above, in the presence of RBCs, particles with aspect ratios approaching unity (spherical shapes) marginate better.

The above findings were also supported by results obtained from two other studies, which provided further evidence that red blood cells play a key role in plasma flow dynamics, with higher hematocrit increasing NP margination within the capillary (Tan et al. 2012; Fullstone et al. 2015). Tan et al. further demonstrated that small, rod-shaped particles have greater binding capacity than spherical ones due to reduced drag and greater contact area (Tan et al. 2013).

The adhesion of NPs with the endothelium during the transit of particles through microvasculature has been extensively investigated using a mathematical modeling approach by Decuzzi and Ferrari et al. (Decuzzi and Ferrari 2006; Van De Ven et al. 2012). They explored the binding of spherical and non-spherical particles under the influence of dislodging hemodynamic forces and adhesive nonspecific or specific interactions at the NP-endothelial cell interface. A force balance between hemodynamic forces, specific receptor-ligand interactions, and non-specific forces (van der Waals, electrostatic, steric) acting on the NP governs the probability of adhesion *P*_*a*_ of NPs to vasculature, expressed as:

12 $$ {P}_a={m}_r{m}_l{K}_a^0{A}_c{e}^{-\frac{\lambda {F}_{dis}}{k_BT{m}_r{A}_c}}, $$

where *m*_*r*_ and *m*_*l*_ are the surface density of receptors on cells and ligands on NPs, respectively; ${K}_a^0$ is the association constant at zero load of ligand-receptor pair; *A*_*c*_ is the area of interaction between the particle and the substrate; *λ* is a characteristic length of the ligand–receptor bond typically on the order of 1 Å; *k*_*B*_*T* is the Boltzman thermal energy; and *F*_*dis*_ is the total dislodging force. Their results suggest that particles with an oblate shape tend to have a higher propensity and strength of adhesion under laminar flow compared to spherical particles.

### Cellular internalization

#### Discrete models

**Fig. 6:**
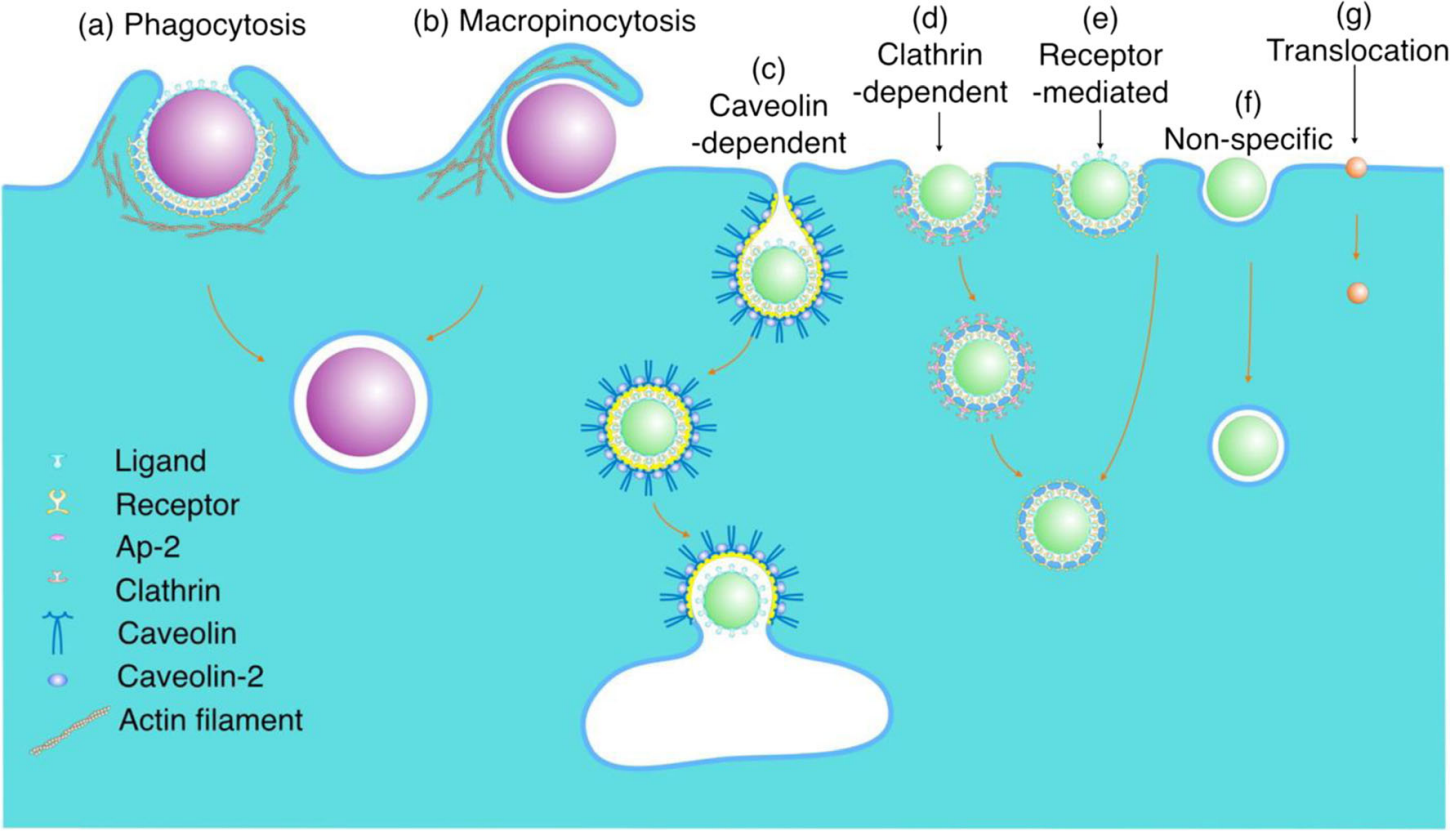
**Cellular internalization pathways.** Phagocytosis (**a**) or micropinocytosis (**b**) may be involved in the internalization of micrometer-sized particles. Caveolin-dependent (**c**) or clathrin-dependent (**d**) endocytosis occurs by receptor-ligand binding leading to the formation of flask-shaped caveolae and clathrin-coated pits, respectively, on the cytosolic side of the cell membrane. Although receptor-mediated endocytosis may also be clathrin- and caveolin-independent (**e**). Non-specific interactions maybe involved in endocytosis of NPs without conjugated ligands (**f**). Small NPs and molecules (<1 nm) may enter the cell by diffusion (translocation) through the plasma membrane (**g**). Reproduced with permission from (Zhang et al. 2015)

Depending upon the size of the particle and its surface chemistry, different pathways may be invoked for cellular internalization of NPs, with receptor-mediated endocytosis (clathrin-, caveolin-dependent and -independent) being the most common pathway for particles in the nano-sized range (Fig. 6) (Zhang et al. 2015). To this end, Gao et al. developed a mathematical model to understand the mechanism of cell membrane wrapping around a spherical or cylindrical particle during receptor-mediated endocytosis (Gao et al. 2005). The model assumes that the particle surface is uniformly coated with immobile surface ligands that are complementary to mobile receptors on the cell membrane surface. Given that cells are much larger than NPs, the problem is simplified by assuming NP interaction with a flat membrane. Following initiation of contact and receptor-ligand binding, the diffusive receptors on cell membrane are drawn towards the site of contact to match receptor density with ligand density, thereby increasing the area of contact over time; this process continues until the area of contact equals the area of the particle. The power balance between the rate of free energy reduction gained from the wrapping process (receptor-ligand binding and membrane cytoskeleton bending) and the rate of energy dissipation consumed during receptor migration yields the particle wrapping time *τ*:

13 $$ \tau ={\left(\frac{\pi R}{2\alpha \sqrt{D}}\right)}^2,\left(\mathrm{for}\ \mathrm{cylindrical}\ \mathrm{particles}\right) $$

14 $$ \tau ={\left(\frac{R}{\alpha \sqrt{D}}\right)}^2,\left(\mathrm{for}\ \mathrm{spherical}\ \mathrm{particles}\right) $$

where *R* is the radius of the particle, *α* is the speed factor, and *D* is the diffusivity of membrane receptors. Their model highlights the importance of particle size in receptor-mediated endocytosis.

Yuan and Zhang developed a model based on the energy balance between receptor-ligand binding energy and work done in membrane wrapping around the particle (Yuan and Zhang 2010). Their model provided analytical expressions for minimal particle size for a given ligand density *ξ*_*l*_ (Eq. 15) and minimal ligand density for a given particle size of radius *R* (Eq. 16) below which receptor-mediated endocytosis cannot occur:

15 $$ {R}_{min}=\sqrt{2B/\left[{\xi}_l\left(\varepsilon +\mathit{\ln}\ {\xi}_0\right)\right]}, $$

16 $$ {\xi}_{l,\mathit{\min}}=2B/\left[{R}^2\left(\varepsilon +\mathit{\ln}\ {\xi}_0\right)\right], $$

where, *ξ*_0_ is the receptor density on cell surface, *B* is the membrane bending modulus, and *ε* is the receptor-ligand binding energy. Their findings suggest that both NP size and ligand density are critical in governing the kinetics of endocytosis, and are important in guiding the rational design of NPs.

In another model, which was experimentally validated using iron oxide NPs and human macrophages, Lunov et al. ignored receptor diffusion on cell membrane surface, and instead employed pit formation followed by membrane wrapping around the spherical particle as the mechanism of receptor-mediated endocytosis (Lunov et al. 2011). By equating the mechanical work performed by cytoskeletal motor proteins for pit formation with the elastic energy of the membrane, the wrapping time *τ* is obtained as:

17 $$ \tau =\frac{4\pi {R}^2\sigma }{p}, $$

where *R* is the particle radius, *σ* is the surface tension of the membrane, and *p* is the power of motor proteins. Their analysis provides quantification of important parameters, including rate of NP uptake, number of NPs uptaken per cell at saturation, and mean uptake time.

Sorrell et al. investigated endocytosis of NPs by cancerous cells using a kinetic model based on a system of differential equations (Sorrell et al. 2014). The model accounts for the rate of change of unbound, bound, and internalized NPs. Key model parameters include association and dissociation rate constants of NP-receptor binding, the recycling rate constant of receptors, and the internalization rate constant of NPs. The results suggested that the rate of NP uptake depends on the number of receptors engaged by the particle.

Decuzzi and Ferrari modified the model by (Gao et al. 2005) assuming a non-constant radius of curvature of the cylindrical particle, i.e., the curvature depends on the particle-cell area of contact and thus varies with time (Decuzzi and Ferrari 2008). As a result, the contribution of the elastic bending energy of membrane wrapping around the particle varies as the area of contact changes. Their model reveals the occurrence of a critical size and aspect ratio for cylindrical particles: for circular cylindrical particles, radius below ~40 nm are energetically unfavorable for internalization, and for elliptical cylindrical particles there exists an optimal range of aspect ratio values, below or above which internalization is incomplete.

### Whole-body biodistribution and clearance

#### Pharmacokinetic models

**Fig. 7:**
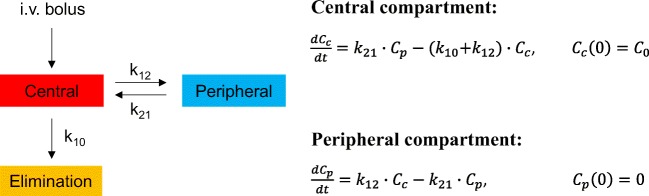
**Schematic of a classical pharmacokinetic model for i.v. bolus administration.** A representative two-compartment PK model with a central compartment and a peripheral compartment is shown. Elimination is restricted to the central compartment. Master equations for the two compartments are also shown, where *C*_*c*_ and *C*_*p*_ are NP concentrations in the central and peripheral compartments, respectively; ***k***_**12**_ and ***k***_**21**_ are the rates of transfer of NPs between central and peripheral compartments, and ***k***_**10**_ is the rate of elimination of NPs from the central compartment. *C*_*0*_ is the concentration in the central compartment at time zero. All mass transfer processes are assumed to follow first-order kinetics, and solution of the coupled ordinary differential equations provides the temporal evolution of NP concentration in the given compartments

For successful clinical translation of nanomedicine, it is important to establish the pharmacokinetics (PK) of NPs, and to this end, mathematical modeling approaches are routinely used; these are referred to as pharmacokinetic models. PK modeling typically operates at the macroscopic (organ) scale and involves a phenomenological description of the ADME (absorption, distribution, metabolism, and excretion) of NPs. PK modeling can be roughly classified into *classical* and *physiological* modeling approaches. The classical PK approach deconstructs the body into a system of compartments, and often contains a central compartment that may be connected via rate constants to one or more peripheral compartments (Fig. 7). The central compartment is a lumped compartment that contains the blood pool of the body and highly perfused organs, like heart, lungs, liver, and kidneys. Similarly, the peripheral compartment is generally formed by lumping the poorly perfused or slowly equilibrating tissues, like fat, bones, and muscle. First-order kinetics is typically assumed for mass transfer between compartments and for elimination from compartments. ODEs are used to describe various PK processes, which are fit to the concentration-time data of the NPs in plasma, urine, or other tissues, to obtain estimates for numerical coefficients and relevant PK parameters like half-life, clearance, volume of distribution, and mean residence time. Being simplistic and empirical (data-driven), such models have great clinical relevance, e.g. dosage regimen design, but their predictive capacity is very limited since they lack an underlying physiological and mechanistic reference (Gerlowski and Jain 1983; Gabrielsson and Weiner 2001; Jones and Rowland-Yeo 2013).

**Fig. 8:**
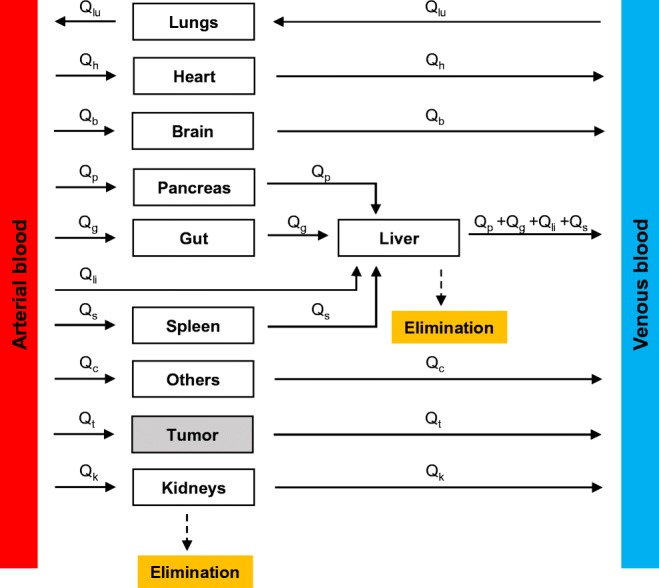
**Schematic of a physiologically based pharmacokinetic model.** The various compartments are connected to the systemic circulation compartments (arterial and venous) via physiological blood flow rates (Q_*i*_). Liver and kidneys are the compartments responsible for clearance. Liver has a dual blood supply as shown (hepatic artery and portal vein). “Others” represents the tissues not explicitly modeled and may include fat, muscle, and bones. Lymph flow is not depicted in this model

This is where physiological PK models fill the gap. Usually referred to as “physiologically based pharmacokinetic (PBPK) models,” they have a similar underlying framework as classical PK models but have anatomically based compartments connected via physiological blood flow rates (Fig. 8). The models are thus parameterized with anatomical and physiological variables (e.g., tissue weights, tissue volumes, blood flow rates (Table 3)) and physicochemical information of the xenobiotics (e.g., blood-tissue partition coefficients), obtained from literature or data fitting (Gerlowski and Jain 1983; Li et al. 2010; Jones and Rowland-Yeo 2013). Each compartment representing a relevant organ or tissue is sub-compartmentalized into vascular, interstitial, and cellular spaces. Based on the physicochemical properties of the xenobiotic under investigation, PBPK models are classified into: i) perfusion or blood flow rate-limited and ii) diffusion or permeability-limited (Khalil and Läer 2011). Perfusion rate-limited models assume that xenobiotic transfer between the vascular space and interstitial space of an organ is not limited by capillary permeability but is only governed by the blood flow rate to the organ. Conversely, permeability-limited models assume capillary permeability to be the limiting factor. When applied to nanomedicine, PBPK models serve to provide a mechanistic description of the concentration time-course of NPs in any given tissue or plasma. Like classical PK, linear kinetics is usually assumed for mass transport across the system and coupled ODEs model the NP concentration in tissues. Because the underlying framework is physiologically meaningful, these models can help with dose extrapolation from animals to humans, or healthy volunteers to diseased patients, based on differences in physiological parameters (Sharma and McNeill 2009; Jones and Rowland-Yeo 2013). They are also valuable in predicting dose adjustments for special populations, like pregnant women (Gaohua et al. 2012) and pediatrics (Hornik et al. 2017). PBPK has also emerged as a critical tool in addressing regulatory questions about the effect of intrinsic (e.g., organ impairment, age, and genetics) and extrinsic (e.g., drug-drug interactions) factors on the PK and pharmacodynamics (PD) of drugs (Huang and Rowland 2012).

**Table 3:** List of a priori physiological parameter values for lab animals and humans for organ- or whole-body-scale models

	Mouse (0.02 kg)			Rat (0.25 kg)			Rhesus monkey (5 kg)			Human (70 kg)		
	Weight (g)	Volume (ml)	Blood flow rate (ml/min)	Weight (g)	Volume (ml)	Blood flow rate (ml/min)	Weight (g)	Volume (ml)	Blood flow rate (ml/min)	Weight (g)	Volume (ml)	Blood flow rate (ml/min)
Brain	0.36	0.48	0.46	1.8	1.2	1.3	90	94	72	1400	1450	700
Heart	0.08	0.095	0.28	1	1.2	3.9	18.5	17	60	330	310	240
Lung	0.12	0.1	≈ C.O.	1.5	2.1	≈ C.O.	33	35.7	≈ C.O.	1000	1170	≈ C.O.
Liver	1.75	1.3	1.8	10	19.6	13.8	150	100	218	1800	1690	1450
Gut	1.5	1.5	1.5	6.3	11.3	7.5	230	230	125	2100	1650	1100
Spleen	0.1	0.1	0.09	0.75	1.3	0.63	8	5.95	21	180	192	77
Pancreas	0.2	0.09	0.27	1	1	1	5	12.5	10.2	80	104	41
Kidneys	0.32	0.34	1.3	2	3.7	9.2	25	30	138	310	280	1240
Muscle	–	10	0.91	–	245	7.5	–	2500	90	–	35,000	750
Fat	–	1.98	–	–	10	0.4	–	154	20	–	10,000	260
Skin	–	2.9	0.41	–	40	5.8	–	500	54	–	7800	300
Blood	–	1.7	–	–	13.5	–	–	367	–	–	5200	–
Plasma	–	1	–	–	7.8	–	–	224	–	–	3000	–
Hepatic artery	–	–	0.35	–	–	2	–	–	51	–	–	300
Portal vein	–	–	1.45	–	–	9.8	–	–	167	–	–	1150
Cardiac output (C.O.)	–	–	8	–	–	74	–	–	1086	–	–	5600

Source: (Davies and Morris 1993; Gabrielsson and Weiner 2001; Peters 2012; Shah and Betts 2012). Lymph flow rates are ~500 times lesser than blood flow rates (Shah and Betts 2012)

Like free drugs, PK modeling of NPs requires quantification of NP concentration (usually expressed as percent of injected dose per gram of tissue (%ID · g^−1^)) over time in plasma, urine, and other tissues. For this purpose, the most commonly employed techniques include plasma/urine radioactivity quantification (Kommareddy and Amiji 2007; Xu et al. 2013), whole-body radioactivity imaging and quantification (e.g. single photo emission computed tomography (SPECT) (Woodward et al. 2007; Chrastina and Schnitzer 2010; Patil et al. 2011; Black et al. 2015; Li et al. 2016; Ming et al. 2017; Dogra et al. 2018), positron emission tomography (PET) (Schluep et al. 2009; Kumar et al. 2010; Phillips et al. 2014; Chen et al. 2016)), fluorescent imaging (Kumar et al. 2010; Vasquez et al. 2011), magnetic resonance imaging (MRI) (Neubauer et al. 2008), inductively coupled plasma mass spectroscopy (ICPMS) (Crayton et al. 2012), and accelerator mass spectrometry (AMS) (Malfatti et al. 2012). The most suitable compartmental model is then fit to the time course of NP concentration obtained using one or more of these experimental techniques. Most in vivo studies (Yu et al. 2012) report a biphasic decline in plasma (or mediastinum (Schluep et al. 2009), a substitute for plasma when imaging techniques are used) concentration of NPs, following intravenous (i.v.) injection. Thus, a two-compartment model is most commonly employed to describe systemic NP disposition (Fig. 7). However, it is important to note that the frequency of experimental data collected can impact the nature of concentration time course, and a biexponential decline will not always be the case. Hence, some studies report using a one compartment model (for monophasic decline) (Sykes et al. 2014b; Dogra et al. 2018). Thus, the selection of the type of compartmental model depends solely on the observed nature of the concentration time course, which in turn is dependent on the method of data collection.

Classical PK approaches have also been employed to study nanomedicine delivery to solid tumors. Sykes et al. (Sykes et al. 2014b) adapted a mathematical model, developed by Schmidt and Dane Wittrup (2009) for antibodies, to predict tumor delivery efficiency of gold NPs. In this one-compartment model, representing the plasma pool of the body, a monoexponential decay function describes the clearance of NPs from blood following i.v. injection. A tumor compartment is linked to the plasma compartment such that monoexponential decay of NP concentration in the blood acts as a forcing function to govern influx of NPs into the tumor. As a simplification, it is assumed that NP influx into the tumor does not influence the monoexponential clearance behavior of NPs in the blood. Once inside the tumor, permeability of tumor vessels governs the extravasation of NPs into the tumor interstitium. Following extravasation, NPs diffuse through the available volume of tumor interstitium, bind or unbind from cell surfaces, and are eventually endocytosed and degraded by tumor cells. With all reactions assumed to follow first order kinetics, the resulting biexponential function describes the NP-associated, dose-normalized, fluorescence concentration time-course (% ID · ml^−1^, percent of injected dose per ml of tumor) in the tumor compartment. The model was then used to predict the influence of plasma clearance rate and tumor cell binding affinity of NPs on their tumor accumulation (represented by concentration-time area under the curve (AUC)). The model can thus be employed as an in silico platform to test NP design configurations for their impact on tumor accumulation of NPs.

The application of the more mechanistic PBPK models in nanomedicine is in only a premature stage to date, and most studies involve the application of PBPK models in their canonical form (Li et al. 2010; Li et al. 2012; Bachler et al. 2013; Li et al. 2013), i.e., models developed traditionally for free drugs. However, fundamental differences between physicochemical characteristics of NPs and free drugs demand modifications to the structure of conventional PBPK models to better fit the purpose of modeling NP disposition kinetics (Riviere et al. 2013).

**Fig. 9:**
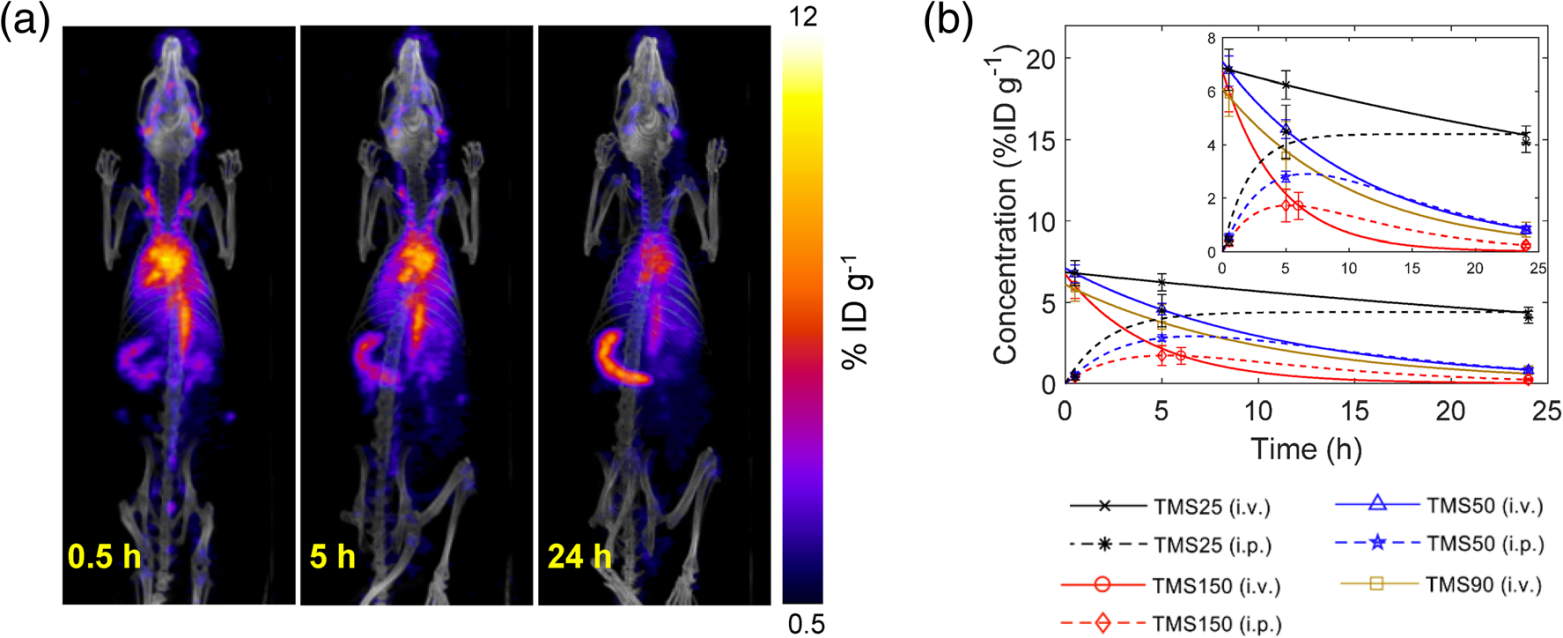
**Imaging-based pharmacokinetics. a** Representative SPECT/CT images of a rat injected with radiolabeled, 25 nm-sized mesoporous silica NPs (MSNs). **b** Concentration kinetics obtained from fitting a phenomenological PK model to concentration versus time data for different types of MSNs injected i.v. or i.p. Reproduced from (Dogra et al. 2018)

*Our group has recently demonstrated the application of PK modeling to disposition kinetics data of mesoporous silica NPs (MSNs) acquired through SPECT/CT imaging* in vivo (Fig. 9) (Dogra et al. 2018). Guided by the data and built on our prior work (Das et al. 2013; Pascal et al. 2013b; Koay et al. 2014; Frieboes et al. 2015), we developed simple master equations in closed form to model the kinetics of MSN biodistribution and clearance with the goal of establishing the structure-activity relationships of MSNs. By systematically varying MSN physicochemical variables (including size, surface charge, and surface coatings) in the therapeutically relevant size range of ~25–150 nm, we examined the effect of size, zeta potential, and surface chemistry on in vivo disposition of hydrodynamically stable, monodisperse, non-targeted MSNs administered via intravenous (i.v.) or intraperitoneal (i.p.) injection. Our analysis showed that (1) smaller MSN size results in a higher systemic bioavailability, irrespective of the route of administration; (2) positive charge favors greater excretion; and (3) surface exposed charged molecules (amines) increase vulnerability to sequestration in liver and spleen. Importantly, a consistent mathematical function between one key PK parameter (AUC_0 − 24 h_; area under the curve―a parameter that evaluates the extent of xenobiotic exposure of organs) and MSN core diameter (*size*) was identified in the form of AUC_0 − 24 h_ = *λ* ∙ size^−*n*^ for systemic circulation and source-like organs (e.g., heart and lungs) in both i.v. and i.p. cases; conversely, for sink-like organs (e.g., spleen and liver), the function identified is AUC_0 − 24 h_ = *λ* ∙ size^*n*^, where *λ* and *n* are fitted parameters. This finding is highly significant because these newly discovered, consistent mathematical functions simplify and facilitate the understanding of the relationships between MSNs’ physicochemical properties and their PK behaviors in vivo.

### Tumor deliverability

#### Hybrid models

We now discuss relevant modeling works that focus on the penetrability of NPs in the tumor tissue. These models prioritize various aspects of NP transport and help understand the relationships between physicochemical properties of NPs and their deliverability to the tumor tissue.

Chauhan et al. developed a mathematical model of tumor vasculature (Chauhan et al. 2012) based on *percolation theory* (Gazit et al. 1995). The model is cast in the form of a two-dimensional percolation network representing tumor vasculature with one inlet and one outlet to study the effect of vascular normalization on tumor delivery efficiency of nanomedicine. The network consists of a series of interconnected nodes representing blood vessel segments, with each segment endowed with vessel wall pores obtained from a distribution of pore sizes. It is assumed that blood flow in the vessels follows Poiseuille’s law,^8^ transvascular fluid exchange occurs according to Starling’s approximation,^9^ and Darcy’s law^10^ governs interstitial fluid transport. Further, the transport of NPs across vessel wall pores is modeled using “pore theory” to determine barriers to diffusion and convection across cylindrical pores. By capturing critical transport phenomena at the tissue scale, this model provides insights into the significance of vascular normalization in cancer nanomedicine. Further, they also studied the effect of NP surface charge on transvascular flux of NPs, and demonstrated the superiority of cationic particles in crossing into the tumor interstitium (Stylianopoulos et al. 2013) using a mathematical model of tumor vasculature based on a previously developed algorithm of tumor-induced angiogenesis governed by vascular endothelial growth factors and fibronectin gradients (McDougall et al. 2006).

Hendricks et al. developed a multiscale kinetic model to study the tumor delivery efficiency of doxorubicin via drug-loaded liposomal formulations (Hendriks et al. 2012). The model consists of a one-compartment PK model of liposome disposition kinetics, integrated with a two-compartment PK model of liposome-released doxorubicin disposition kinetics, in plasma. The PK models are connected to a physiologically based tumor model that incorporates transvascular flow, interstitial transport, and cellular uptake of liposomes and free drug. Their model reveals strong dependence of delivery efficiency on liposomal PK and tumor vascular permeability to liposomes, thereby highlighting the significance of these patient-specific parameters in determining the success of nanotherapy.

**Fig. 10:**
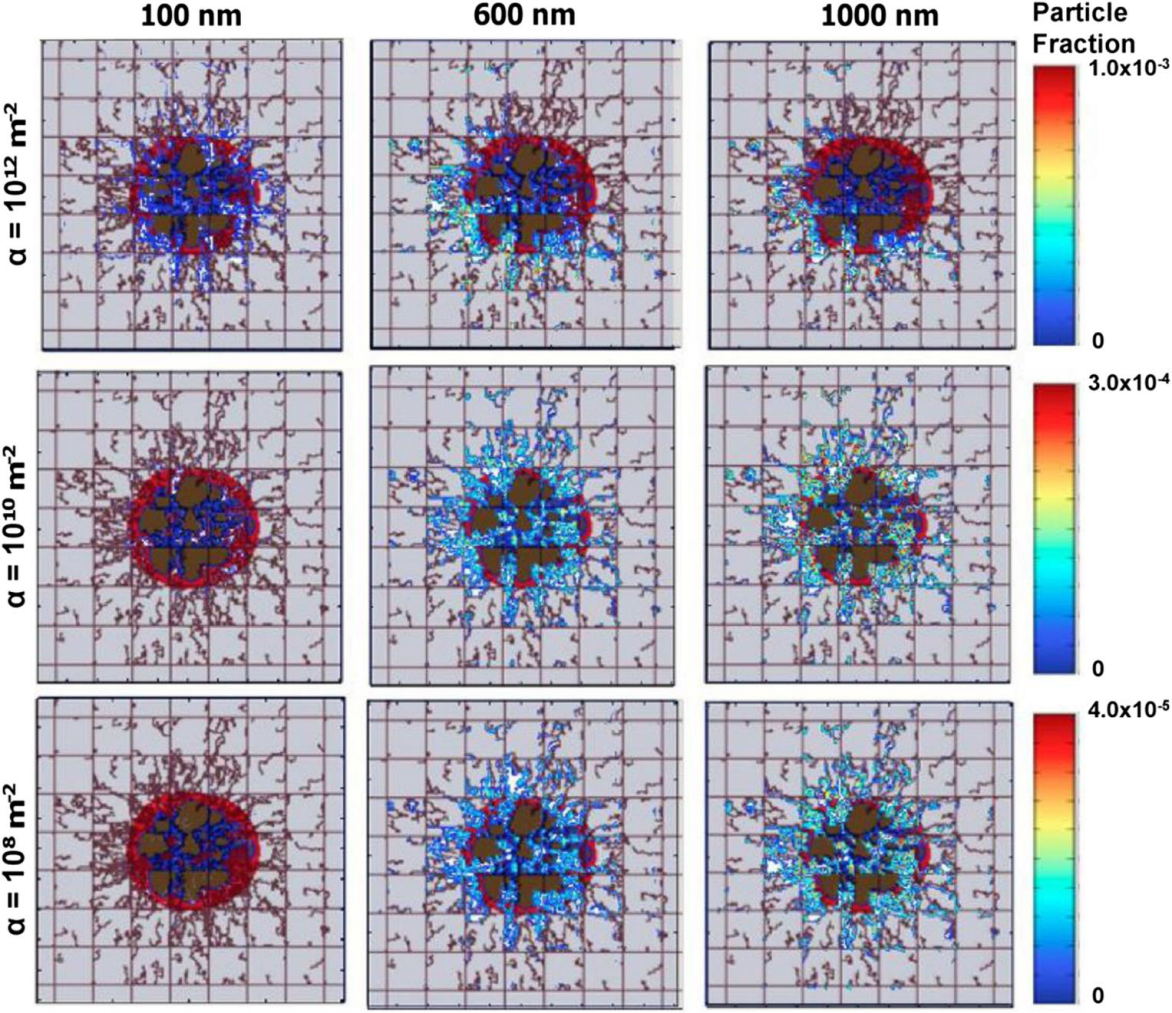
**Model predictions of NP tumor delivery efficiency.** A multiscale tumor growth model is used to predict the delivery efficiency of nanocarriers of different sizes (100 nm, 600 nm, 1000 nm) and at different levels of intratumor vasculature receptor expression (α) 100 min after injection on day 18 of tumor growth. Reproduced with permission from (Frieboes et al. 2013)

Frieboes at al. developed a computational model to predict accumulation of NPs in tumor vasculature (Frieboes et al. 2013). It combines a previously developed two-dimensional model of tumor growth and angiogenesis (Anderson and Chaplain 1998; Cristini et al. 2003; McDougall et al. 2006; Macklin et al. 2009; Wu et al. 2013) with a mesoscopic model of NP adhesion to the tumor neovasculature. Tumor dynamics are primarily governed by cell division, cell death, cell migration, and cell-cell and cell-extracellular matrix adhesion. These processes are accounted for in the tumor growth compartment as a mathematical model based on conservation principles (mass, momentum) and transport phenomena (diffusion, convection). This tumor growth model is then coupled with a model of tumor-induced angiogenesis based on tumor angiogenic factor (TAF) gradients. This component accounts for tumor blood flow, non-Newtonian effects, vascular leakage, and vasculature remodeling due to shear stress and mechanical stress because of a growing tumor, thus simulating a pathophysiologically relevant scenario to test neovascular accumulation of systemically injected NPs based on a mesoscopic mathematical formulation (Eq. (12)) (Decuzzi and Ferrari 2006). Thus, by integrating the evolution of a growing tumor with NP-cell interactions, the model demonstrates the dependence of vascular accumulation of NPs based on the tumor growth stage, in addition to the importance of NP vascular affinity in controlling total accumulation of NPs and their spatial distribution inside a tumor (Fig. 10).

Hauert et al. (2013) modeled a representative section of tumor tissue to study the design characteristics of targeted NPs responsible for binding site barriers (Fujimori et al. 1990), and proposed guidelines to overcome such barriers. The modeled section represents a hypoperfused, near-necrotic region of the tumor where NPs extravasating from the microvessels diffuse into surrounding tumor tissue, and may bind to cell surfaces and subsequently be internalized. Reaction-diffusion kinetics thus forms the basis of such a model, and is represented by the following biochemical reaction: ${NP}_F+R\overset{k_a,{k}_d}{\leftrightarrow }C\overset{k_i}{\to }{NP}_I+R$, where *NP*_*F*_ represents free NPs, *R* refers to cell-surface receptors, *C* are the NP-receptor complexes, *NP*_*I*_ represents internalized NPs, and *k*_*a*_, *k*_*d*_, and *k*_*i*_ are the association, dissociation, and internalization rate constants, respectively. This system has been modeled using both deterministic and stochastic approaches. The deterministic modeling approach is comprised of coupled reaction-diffusion PDEs governing the spatiotemporal evolution of species of interest (*NP*_*F*_, *R*, *C*, *NP*_*I*_) in a one-dimensional domain. The stochastic modeling approach discretizes the spatial domain into cubes with side *S*, assuming a well-mixed volume in each unit. NPs diffuse across these sub-volumes while interacting with surface receptors (potentially forming complexes and undergoing internalization), with each event being governed by a dynamic probability. Unlike the deterministic model, the stochastic model captures the randomness and fluctuations of a biochemical system; however, the former is a better choice for systems with large populations and relatively small fluctuations, since the SSC might be computationally expensive for such systems. Thus, in this work the authors used the deterministic model to simulate different experiments, and only validated key findings using the stochastic model. Their work suggests that delaying the binding of NPs to target cells could avoid binding site barriers on the tumor periphery and allow them to penetrate deeper into the tumor tissue, which can be achieved by engineering NPs to avoid premature cellular uptake.

Stapleton et al. developed a mathematical model to specifically investigate the transport of liposomes in solid tumors through the EPR effect (Stapleton et al. 2013). The model was formulated as a differential equation, and accounts for the transvascular and interstitial convective flux to determine the rate of liposome accumulation in the tumor interstitium. The extravasation of NPs from the vessels is primarily driven by the difference between microvascular pressure and interstitial fluid pressure, while interstitial transport is driven by an interstitial pressure gradient estimated by Darcy’s law. The model was validated against experimental data and predicted inter-subject and intra-tumoral variations in the EPR effect based on biophysical properties of the tumor microenvironment.

Sykes at al. performed Monte Carlo simulations to study diffusion of NPs through tumor extracellular matrix (ECM) (Sykes et al. 2016), based on the approach of (Stylianopoulos et al. 2010). They modeled the tumor ECM in three-dimensions as an anisotropically oriented network of collagen fibers (the most abundant protein in ECM) to study the mobility of NPs through matrices of different collagen densities. Collagen fibers were approximated as immobile cylinders and NP-fiber collision was assumed to be elastic. NP movement was simulated as a discrete random walk following the Stokes-Einstein relation for diffusion of spherical particles in a fluid with low Reynolds number. They also modeled in 2D the microscopic collagen matrix pores to study the effect of NP size and matrix pore size on the frequency of NP-fiber collisions to provide a mechanistic explanation to the results of the 3D model. In this model, a pore was represented by a square bounded by collagen fibers, a NP underwent 2D Brownian motion inside the square, and particles were tracked for their collisions with the wall (fibers). Their models helped elucidate the mechanisms underlying particle size-dependent retention of NPs in clinically relevant tumor conditions.

### Nanotherapy efficacy and toxicity

#### Pharmacodynamic models

**Fig. 11:**
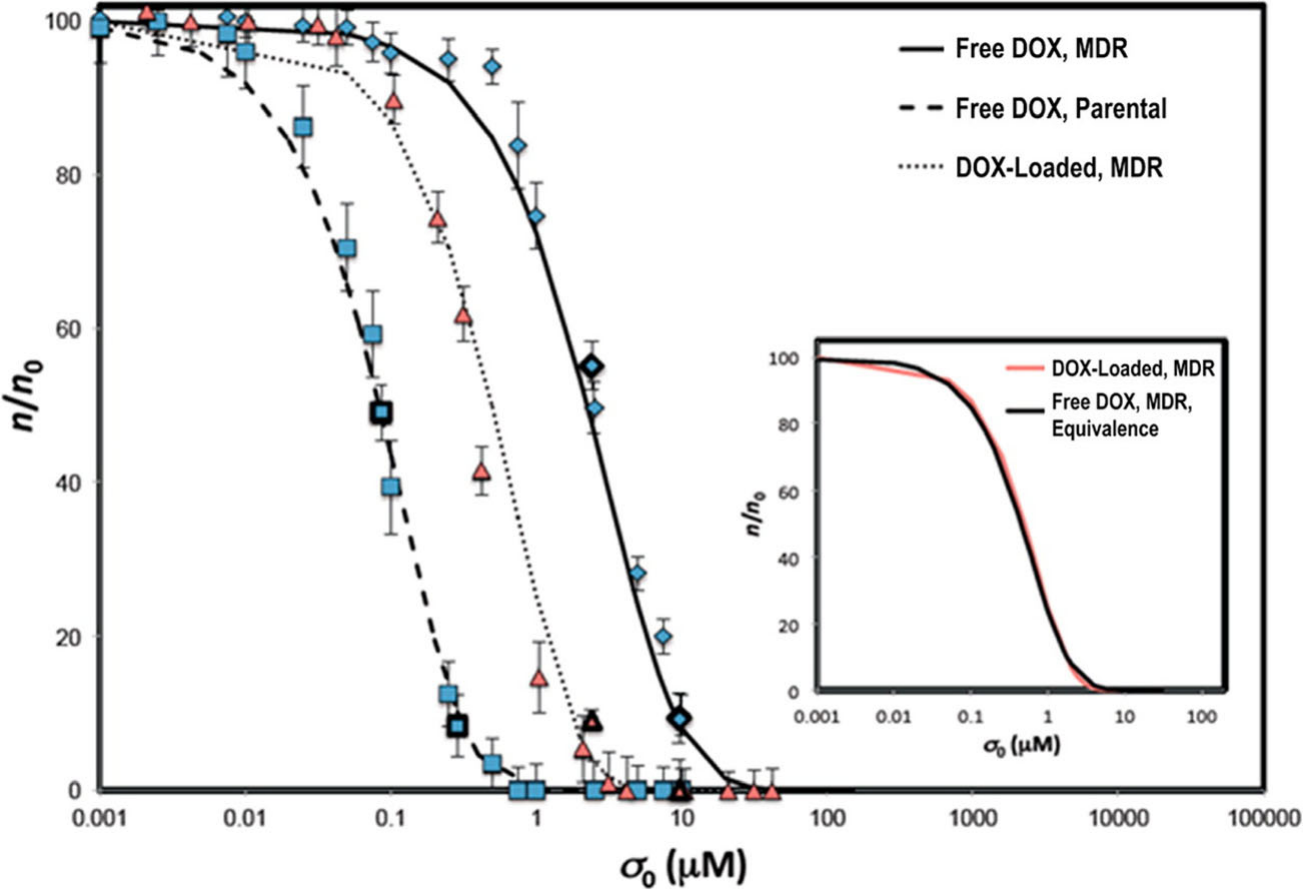
**Predictions of dose-response curve from a mathematical model.** Numerical solutions of the model are tested against experimental observations of the fraction of viable cells 24 h after incubation with free-doxorubicin and doxorubicin-loaded NPs at variable drug concentrations. NP-mediated delivery causes a left-shift (lowered drug IC_50_) in dose-response curves of multi-drug resistant hepatocellular carcinoma cells. Reproduced with permission from (Pascal et al. 2013a)

We now discuss the modeling approaches employed to investigate nanotherapy efficacy, which is the ultimate determinant of success of cancer nanomedicine. (Pascal et al. 2013a) evaluated the cytotoxicity of NP-loaded doxorubicin to hepatocellular carcinoma cells using a mathematical model based on cell and drug mass conservation. The model is based on the hypothesis that cell death rate is a function of the total amount of drug taken up by the cells over time, which is represented by the following equations that predict the asymptotic behaviors of cell and drug concentrations for short and long drug exposure times (*λn*_0_*t*), respectively:

18 $$ \Big\{{\displaystyle \begin{array}{c}\frac{n}{n_0}\approx 1-\frac{1}{2}{\lambda}_A{\sigma}_0\lambda {n}_0{t}^2\\ {}\frac{\sigma }{\sigma_0}={e}^{-\lambda {n}_0t}\end{array}},\kern0.5em \left(\lambda {n}_0t\ll 1\right) $$

19 $$ \Big\{{\displaystyle \begin{array}{c}\frac{n}{n_0}\sim {e}^{-{\lambda}_A\left({\sigma}_0-{\sigma}_{\infty}\right)t}\\ {}\sigma \approx {\sigma}_{\infty}\end{array}},\kern0.5em \left(\lambda {n}_0t\gg 1\right) $$

where, *n*, *n*_0_ are the cell concentrations at time *t* and *t* = 0, respectively, *σ*, *σ*_0_, *σ*_∞_ are the drug concentrations at time *t*, *t* = 0, and *t* = ∞, respectively, *λ*_*A*_ is the specific cell death rate, and *λ* is the specific drug uptake rate by cells. Eq. 18 implies that cells initially consume drug, thus decreasing drug concentration over time at an exponential rate *λn*_0_. As the drug concentration reaches a constant value *σ*_∞_, cells begin to die. At large times (Eq. 19), cells die exponentially, with death rate *λ*_*A*_(*σ*_0_ − *σ*_∞_) linearly proportional to the total amount of drug (*σ*_0_ − *σ*_∞_). Analysis and predictions of this model, validated with in vitro cell viability assays, demonstrate the superiority of drug delivery using NPs over free-drug delivery (left shift in dose response curve in Fig. 11), primarily because NP-based delivery allows cells to uptake drug at a higher rate, thereby enhancing the rate of cell death. This model was extended further to incorporate the effect of spatial heterogeneities of diffusion and perfusion in solid tumors on therapy efficacy, and a closed-form solution was derived for the time-dependent drug concentration and tumor volume equations, given as the fraction *f*_kill_ of viable tumor killed following nanotherapy (Hosoya et al. 2016; Wang et al. 2016; Brocato et al. 2018):

20 $$ {f}_{\mathrm{kill}}=\frac{F{\lambda}_k}{2{V}_{T,0}}{t}^2 $$

where *F* is the flux of drug from the NPs, *F* is a function of NP size, *λ*_*k*_ is the cell death rate per unit cumulative drug concentration, and *V*_*T*, 0_ is the initial tumor volume. Note that there is a quadratic increase in treatment efficacy (represented by *f*_kill_) with time. The model predicts faster and greater tumor death when treated with nanocarriers than with free drug, in excellent agreement with experimental observations under various conditions. In particular, using this model and by linking measured tumor growth with NP distribution, Brocato et al. found that treatment efficacy increases exponentially with increased NP accumulation within the tumor, highlighting the importance of optimizing the delivery efficiency of NPs to the tumor (Brocato et al. 2018).

Stylianopoulos et al. further developed their tumor delivery model (Stylianopoulos et al. 2013) to incorporate controlled release of drugs from NPs and their binding and internalization into tumor cells in order to study efficacy of NP-delivered chemotherapy on cancer cells (Stylianopoulos et al. 2015). They mathematically formulated the interstitial processes of drug release from NPs, drug diffusion, cell surface bonding, and cellular internalization in the form of coupled PDEs in order to model the evolution of internalized drug concentration and compare the cell-kill induced by a two-stage and a multi-stage nanoparticulate system with conventional chemotherapy. The model explored the effects of NP size, drug release kinetics, and binding affinity on therapy efficacy, and demonstrated the superiority of multistage nanocarriers over two-stage NPs. Papageorgis et al. used the same model to mechanistically understand the effect of Tranilast-induced reduction of mechanical stresses in tumors on improved efficacy of nanotherapeutics, thereby proposing the repurposing of tranilast as mechanical-stress alleviating agent (Papageorgis et al. 2017).

Leonard et al. (2016) employed the model developed by Frieboes et al. ((Frieboes et al. 2013) discussed previously) to investigate the efficacy of macrophage-encapsulated NP albumin-bound-paclitaxel in simulated patient breast cancer liver metastasis. Their model demonstrates the potential of this novel formulation for clinical translation based on the histological evidence of tumor biopsies. Miller and Frieboes employed the same model to study the effects of vasculature-induced heterogeneity and drug strength (IC_50_) on therapy efficacy of drug-loaded gold NPs (Miller and Frieboes 2018). The results indicate that in the case of drugs with high IC_50,_ the intra-tumoral vascular density should be above a threshold for optimal transport of nanocarriers for satisfactory efficacy, while efficacy depends less strongly on vessel density for NPs loaded with drugs with lower IC_50_.

A relatively less studied aspect that can potentially stall nanomedicine from clinical translation is NP-induced toxicity. Recently, a multiscale hybrid model was developed by Laomettachit et al. to assess NP toxicity in human liver (Laomettachit et al. 2017). The model uses a standard whole-body PBPK framework to predict hepatic accumulation of administered NPs, and feeds the whole-body model-derived information into a tissue scale “cell-response model” of liver tissue. The cell-response model accounts for cell division and cellular uptake of NPs to predict the impact of NPs on hepatocyte viability. This multiscale platform demonstrates the dose-dependent toxicity of titanium dioxide NPs in liver, i.e., the tissue damage from a low dose of NPs is negligible and reversible due to compensation by cell proliferation, while high exposure can cause irreversible tissue damage unless a large fraction of cells undergoes cell division to renew the damaged tissue mass.

## Conclusions

Cancer nanomedicine has been inspired by Paul Ehrlich’s notion of the “magic bullet”, which visualizes the design of therapeutic agents that selectively attack diseased cells while sparing the healthy ones. Due to the advances in nanotechnology, progress has been made towards specifically delivering cytotoxic agents to cancerous cells, despite the complexities inherent to a cancerous system. Although much of this technology still remains to be translated into the clinic, the investigation to understand and overcome the often-limited performance of nanomedicine-mediated cancer therapy continues. In a nutshell, the challenges associated with successful delivery of nanomedicine to the cancerous tissue range from sub-microscopic NP-protein interactions to microscopic NP-cell interactions, leading to an emergent behavior at the macroscopic scale in the form of whole-body biodistribuiton and clearance from the body. It is imperative to study these interactions in isolation, as well as in unification, to better understand the fundamental principles that lie underneath the problem. Moving forward, the intergration of mathematical modeling with experimental investigation of NP kinetics, efficacy, and toxicity in order to better under the structure-activity relationships of nanocarriers for their clinical translatability will become increasingly important. This review has explored many of the mathematical modeling approaches developed in the field of cancer nanomedicine that seek to expand our capability to fully explore the nano-bio interactions. In the future, we believe nanomedicine will bring notable improvements in patient survival and quality of life.

## Footnotes

1 Microscopic particles with aerodynamic diameter < 100 nm.

2 Adsorbed with plasma proteins of the complement system (opsonins) that cause recognition of xenobiotics by the immune system.

3 Volume percentage of red blood cells in blood.

4 Lateral drift of particles towards the vessel well.

5 Stokes-Einstein equation provides the diffusion coefficient for spherical particles in low Reynolds number. ${D}_m=\frac{k_BT}{6\pi \eta r}$, where *k*_*B*_ is Boltzmann’s constant, *T* is the absolute temperature, *η* is dynamic viscosity, and *r* is the radius of NP.

6 Magnetized NP is substituted by an equivalent point dipole with an effective dipole moment *m*_*p*, eff_ such that the magnetic force *F*_*m*_ on the dipole is, *F*_*m*_ = *μ*_*f*_(*m*_*p*, eff_ · ∇)*H*_*a*_, where *μ*_*f*_ is the permeability of the fluid, and *H*_*a*_ is the external magnetic field intensity.

7 The drag *F*_*d*_ on a spherical object of radius *r*, moving through a viscous fluid of viscosity *η* at a relative velocity *v*, is, *F*_*d*_ =  − 6*πηrv*.

8 ${Q}_{vasc}=-\frac{\pi {d}^4}{128\mu}\frac{\Delta {p}_v}{\Delta x}$ where *Q*_*vasc*_ is the blood volumetric flow rate in a vessel of length Δ*x* and diameter *d*, for vascular pressure difference Δ*p*_*v*_ and blood viscosity *μ*.

9 *Q*_*transv*_ = *L*_*p*_*S*(*p*_*v*_ − *p*_*i*_) where *Q*_*transv*_ is the volumetric fluid flow rate across a vessel wall of hydraulic conductivity *L*_*p*_, vessel surface area *S*, and interstitial fluid pressure *p*_*i*_.

10 ${Q}_{inter}=-{k}_t{A}_c\frac{\Delta {p}_i}{\Delta x}$ where *Q*_*inter*_ is the volumetric fluid flow rate in interstitial space with hydraulic conductivity *k*_*t*_, cross-sectional area *A*_*c*_, tissue length Δ*x*, and interstitial pressure difference Δ*p*_*i*_.
